# Modern pollen data from the Canadian Arctic, 1972–1973

**DOI:** 10.1038/sdata.2017.65

**Published:** 2017-05-16

**Authors:** Harvey Nichols, Susann Stolze

**Affiliations:** 1 Department of Ecology and Evolutionary Biology, University of Colorado, Boulder, Colorado 80309, USA; 2 4255 Chippewa Drive, Boulder, Colorado 80303, USA; 3 Institute of Arctic and Alpine Research, University of Colorado, Boulder, Colorado 80303, USA; 4 Department of Geology and Geological Engineering, Colorado School of Mines, Golden, Colorado 80401, USA

**Keywords:** Boreal ecology, Phenology, Biogeography, Pollen

## Abstract

This data descriptor reports results of a 1972–73 baseline study of modern pollen deposition in the Canadian Arctic to originally aid interpretation of Holocene pollen diagrams from that region, especially focussed on the arctic tree-line. The data set is geographically unique due to its extent, and allows the assessment of the effects of modern climate change on northern ecosystems, including fluctuations of the a arctic tree-line. Repeated sampling was conducted along an interior transect at 29 sites from the Boreal Forest to the High Arctic, with five additional coastal sites covering a total distance of 3,200 km. Static pollen samplers captured both local pollen and long-distance pollen wind-blown from the Boreal Forest. Moss and lichen polsters provided multi-year pollen fallout to assess the effectiveness of the static pollen samplers. The local vegetation was recorded at each site. This descriptor provides information on data archived at the World Data Center PANGAEA, which includes spreadsheets detailing site and sample information as well as raw and processed pollen data obtained on over 500 samples.

## Background & summary

Arctic and subarctic ecosystems are highly sensitive to climate variability and may be the most responsive of any major biomes to paleo- and modern climatic changes^1,2^ due to arctic amplification mechanisms^3^. Paleoecological data show that high-latitude biomes such as the arctic tree-line respond strongly to global warming and cooling^1,4–7^. For instance, it has been shown that a ~400 km northward tree-line advance during the mid-Holocene occurred in response to an estimated ~4 °C mean summer regional warming^5^.

Holocene palynological studies demonstrating changes in the location of the arctic tree-line were pioneered in North America by H. Nichols^1,4,8–12^ and extended by Ritchie^6,13^. These studies required modern pollen data to understand temporal changes in fossil pollen spectra and pollen-vegetation relationships. At the time, surface pollen data available from only few Canadian Holocene fossil sites did not sufficiently explain the arctic-subarctic pollen stratigraphy. To overcome the paucity of surface pollen data, an extensive sampling program in the Canadian Arctic was conducted, involving three expeditions in 1972–73 funded by the National Science Foundation. More recently, emphasis in modern pollen deposition in northern North America largely shifted towards surface lake sediments^14–22^ due to the fact that peat sequences could show discontinuities or disturbances^21^. However, peat stratigraphy and rates of growth provide significant paleoclimatic evidence of airmass dominance and moisture balance that complement lake stratigraphies^5^, which can be seriously disturbed due to anchor-ice flotation^23^.

**Figure 1:**
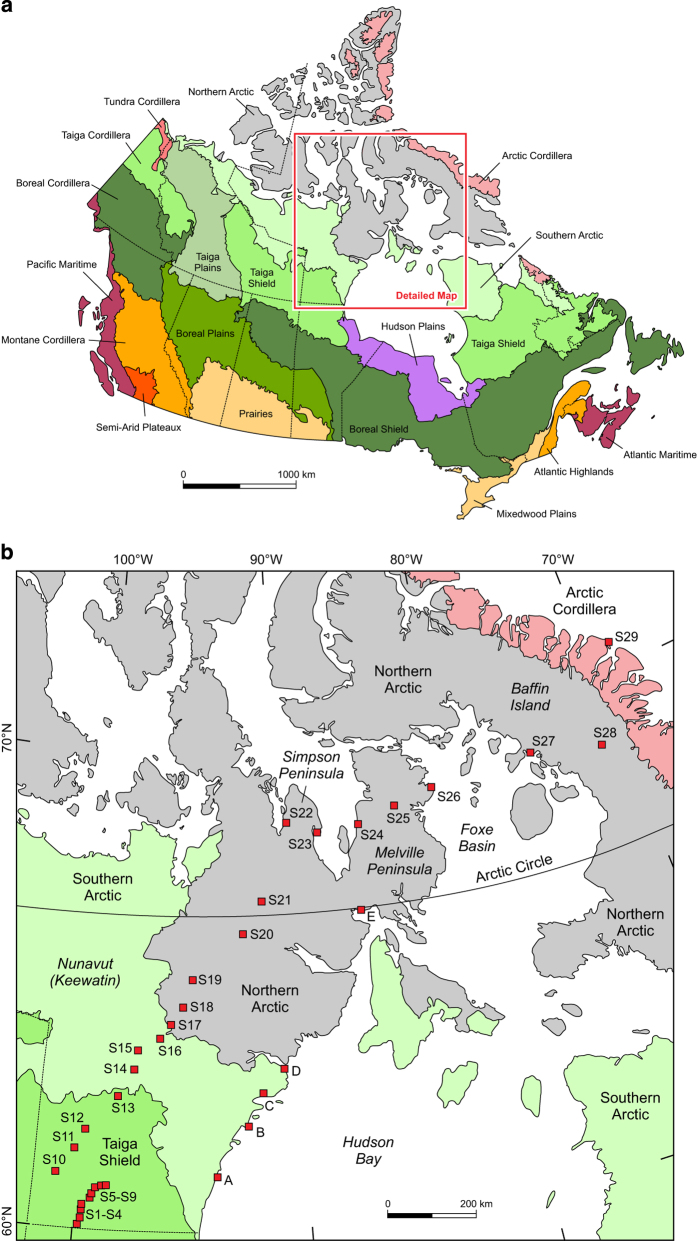
Locations of modern pollen collection in the Canadian Arctic in 1972–73. (**a**) Map of Canada showing ecozones^30^ and the sampling region. (**b**) Detailed map of the sampling region. Red squares indicate the sampling locations of the interior transect (sites S1‒S29) and the coastal transect (sites A‒E).

This data descriptor presents for the first time the complete results of this baseline study on surface pollen deposition in the Canadian Arctic using Tauber and Benninghoff samplers as well as moss and lichen polsters. The moss polster data was previously published in a study on modern pollen deposition and Holocene paleotemperature reconstructions in central northern Canada^24^ and the findings were used in the interpretation of arctic fossil palynological data^5,25^. Two summer seasons of annual pollen deposition were recovered from a 2,000 km transect of 29 sites in interior Keewatin (Nunavut) from the arctic tree-line to high arctic Baffin Island, with additional five sites sampled along 700 km of the Hudson Bay western coast (Fig. 1). A total of 135 pollen samples were collected using unpowered standard Tauber samplers^26^ and experimental Benninghoff samplers^27^ over a two-year period. A total of 287 moss and lichen polsters near the static pollen collectors were sampled to compare the trap-sampled annual pollen rain with the multi-year pollen deposition contained within these natural pollen collectors. The vegetation and environmental setting at each site was recorded to assist in the interpretation of the pollen data. The pollen samples were studied microscopically to determine absolute and relative pollen values. Intermediate pollen sums were recorded to evaluate the reliability of the final counts. Rare exotic pollen types such as pine and spruce pollen were documented by scanning additional large areas of microscope slides at low magnification, because such exotic pollen types from distant sources can provide important paleo-wind information^25^. The data described in the present contribution represent a geographically unique baseline data set for the interpretation of Holocene pollen stratigraphy^5^. Together with modern pollen studies using polster and Tauber samplers as well as surface lake sediments conducted across the arctic tree-line and tundra environments in Finnish Lapland^28–30^ and North America^14–16,20,22^, the present study provides a benchmark for studies of climate change impact on arctic plant communities.

## Methods

### Research design

An interior transect and a subsidiary coastal transect totalling 34 modern pollen sampling sites were established from the interior of Nunavut (formerly Keewatin) ~60°N to north east Baffin Island at ~70°N and along the west coast of Hudson Bay (Fig. 1), using wintertime ski- and summertime float-planes. The 2,000 km long interior transect of 29 sites (S1‒S29; Fig. 1; Table 1) began near the northern woodland edge of the Taiga Shield^21^ and crossed the arctic tree-line ecotone at ~60°N. This transect continued at close sampling intervals from that ecotone, northwards through the southern arctic^31^ (Fig. 1). The transect ran north-eastwards through the ‘Barren Grounds’ and the cold semi-desert of the northern Arctic^31^ to the Arctic Cordillera^31^ of Baffin Island at ~70°N, facing Greenland across Davis Strait (Fig. 1). The interior transect was arranged so that the meteorological stations at Ennadai Lake and Baker Lake were included, as well as the weather stations at Distant Early Warning Line (DEW-Line) radar sites on the coast of the Arctic Ocean: Pelly Bay, Mackar Inlet, Sarcpa Lake, Hall Beach, Dewar Lakes, and Longstaff Bluff. At the far northern end of the interior transect, the coastguard long-range navigation (LORAN) station of Cape Christian at ~70°N, which is no longer in service, was also chosen for its weather station.

In the southern part of the interior transect the sample sites were ~15 km apart in the northern woodland and across the arctic tree-line ecotone, chosen at close intervals to register what were expected to be rapid changes in forest pollen deposition with increasing northward latitude and distance from the tree-line ecotone. These changes would include rapidly declining *Pinus* and *Picea* pollen numbers, which would prove most useful when estimating distance from the arctic tree-line in fossil palynological studies. In the southern part of the study region, particular attention was focused on monitoring the fertility of isolated spruce tree ‘islands’ as indices for a changing climate. A 1993 re-examination of those same relict trees showed them to be fertile, presumably due to current polar warming^32^. Farther away from the woodland–tundra boundary, the site locations were chosen ~85 km apart since abrupt changes in the ‘exotic’ long-distance forest pollen were not expected far out in the tundra.

A subsidiary transect of five sampling sites was set up along the west coast of Hudson Bay, stretching over ~700 km (Fig. 1; Table 1). These sites included Eskimo Point (site A), Whale Cove (site B), Rankin Inlet (now Arviat; site C), Chesterfield Inlet (site D), and Repulse Bay (site E).

Monitoring of the pollen deposition at these 34 locations was designed to include two flowering seasons from late winter in 1972 through late summer 1973. Repeated sampling was considered to be important to provide statistically meaningful pollen data and to collect sufficient data in case of sampler loss or other logistical issues during the first season. To establish modern pollen-vegetation relationships, recording of vegetation was planned and made in writing, by color 35mm slides, audio tape, and Super 8 film recording for each site.

### Setting out the collectors and sample collection

This research program was based on H. Nichols’ three expeditions in 1972 and 1973, which were carried out to set up the pollen samplers and to collect the annual pollen rain along the interior and coastal transects. These expeditions commenced at the base station in Churchill, Manitoba, and each totaled a travel distance of ~3,200 km by small plane for a total of ~9,600 km. Transport was by single-engine aircraft (Turbo Beaver, Otter, or Aztec) using wheel-skis for landing on frozen lakes and gravel strips in late arctic winter (early June). The planes used in summertime were equipped with pontoons for landing on interior lakes and coastal sites in late arctic summer (August). The coordinates of the locations were recorded by the pilots using the existing ‘pre-GPS’ aviation maps and the locations were verified using satellite imagery (Google Earth). The three expeditions took place from 6‒18 June 1972, 3‒14 August 1972, and 3‒22 August 1973, starting from and returning to Churchill, Manitoba.

During the first expedition in early June 1972 prior to local arctic summer flowering, Tauber and Benninghoff samplers were installed and polster samples were collected, beginning in the southern part of the interior transect at the meteorological station at Ennadai Lake (S10). Due to ice decay, lake landings were not feasible at any location farther south at that time, so that sites S1‒S9 at Nueltin Lake were left for the expedition in August 1972. The June expedition proceeded northwards to site S19 (Fig. 1; Table 1), which is over 100 km beyond the Inuit settlement of Baker Lake. The unusually lengthy winter of 1972 left deep ridged snow cover on lakes north of site S19 ~120 km north of Baker Lake (64°N) and made increasingly difficult landings and take-offs by the Turbo Beaver plane, exacerbated by large-scale advection fog which the visual-flight-regulation (VFR) aircraft was forbidden by regulation to overfly. Therefore, on 15 June 1972, the expedition broke out east to install samplers on the west coast of Hudson Bay, going north from Chesterfield (site D) and Repulse Bay (site E) to Hall Beach (S26) in the Foxe Basin on the Arctic Ocean coast (Fig. 1; Table 1). Late-lying snow cover and bad weather forecasts prevented the expedition crossing the mountains of central Baffin Island using the VFR-regulated Turbo Beaver plane. Thus setting out the samplers in this high arctic region was delayed until August 1972.

On the second expedition in August 1972, the pollen rain from the first flowering season was collected from sites S10‒S14, S16‒S19, S26, and sites A‒E. In addition, more moss and lichen samples were collected (Table 1) and additional static collection devices were installed along the planned interior transect that was previously curtailed in early June by unusual winter conditions. Sample collection commenced along the coastal transect on the west coast of Hudson Bay, at Eskimo Point (site A), proceeding north to Repulse Bay (site E), and then Hall Beach (S26) on the mainland coast facing Foxe Basin (Arctic Ocean; Fig. 1). The expedition continued from Hall Beach (S26) eastwards to the northern end of the interior transect on Baffin Island to install pollen samplers at Longstaff Bluff (S27), Dewar Lakes (S28), and the northernmost site at Cape Christian (S29), facing Greenland (Fig. 1). From there, the aircraft turned west along the Arctic Ocean coast to install samplers on Melville Peninsula and Simpson Peninsula at Sarcpa Lake (S25), Mackar Inlet (S24), Keith Bay (S23), and at Pelly Bay (S22; Fig. 1). The expedition then turned south into the high arctic tundra interior of Keewatin to set up pollen collectors from north to south beginning at Sandspit Lake (S21), Esker Lake (S20), unnamed lake (S19), and Tehek Lake (S18). Continuing south, pollen samples from the first flowering season were then collected from sites first emplaced in late winter of June 1972: Baker Lake (S17), unnamed lakes (S16 and S14), Yathkyed Lake (S13), unnamed lakes (S12 and S11), and Ennadai Lake (S10). Because the unnamed lake S15 was too shallow for safe water landing by float plane, in comparison to the frozen lake landing using skis in June 1972, the collectors installed in June 1972 had to be abandoned. Therefore, a new site near the original site S15 was chosen to install a new Tauber sampler in August 1972. The exact location of the new site relative to the original site S15 could not be determined in ‘pre-GPS’ days. During the August 1972 expedition, the interior transect was extended further southeast to Nueltin Lake to cover the woodland-tundra transition. Sampling at close intervals (15 km apart) commenced at the southern end of Nueltin Lake and proceeded north to Sealhole Lake (sites S1‒S9).

The last expedition in August 1973 sampled the pollen rain from the flowering season of 1973. The first leg of the expedition using a pontoon Otter aircraft set off northwards along the west coast of Hudson Bay, sampling sites Eskimo Point (A), Rankin Inlet (C), Repulse Bay (E), and Hall Beach (S26). Logistical considerations associated with early lake freezing led to the samples being collected at Whale Cove (B) and Chesterfield (D) by another pilot and delivered to the expedition base station in Churchill. The expedition proceeded immediately as far north as possible using the alternate Aztec aircraft using wheels to land on gravel airstrips to avoid an early onset of winter conditions, sampling Longstaff Bluff (S27), Dewar Lakes (S28), and Cape Christian (S29) on Baffin Island. Turning southwest, sites S25, S24, and S22 on Melville Peninsula and Simpson Peninsula were collected (Fig. 1). No samples were retrieved from Keith Bay (S23) due to massive frost cracking in the old landing strip and unsafe landing conditions for the Aztec aircraft. From there, the original charter using the Otter float plane resumed and headed south into the interior of Keewatin, sampling from north to south the sites S21‒S1 (Fig. 1; Table 1).

### Tauber samplers

At each sampling site, three Tauber samplers (TAU1‒TAU3) were emplaced along a ~40-m long transect from the lakeshore to the nearest topographic high to study variability in pollen rain at small spatial scale and to provide a back-up in case of sample loss. Samplers were spaced ~20 m apart perpendicular to the lakeshore. In the southern portion of the interior transect, the first Tauber sampler installed near the lakeshore was placed next to dwarf spruce tree islands at sites S1‒S4 and S8‒S13 (Fig. 1) to examine their fertility by capturing any pollen produced by these diminutive tree islands. These dwarf mature trees were living *Picea mariana* of up to ~2 m in height. The second Tauber sampler was positioned on the sloping land rising from the lakeshore to collect regional pollen rain and any pollen from the tree islands. The third sampler was set out farther away on the nearest high point to minimize any influx of spruce pollen from the tree islands. The absence of cones and the tree island pollen counts showed them not to be fertile at that time.

The Tauber samplers are standard unpowered pollen collectors that collect atmospheric pollen deposited by gravity, by horizontally carried pollen grains, and by particles scavenged by precipitation^26^. The aerodynamically designed top avoids sampling anomalies such as edge effects and sampling shadows^33^. Although the pollen depositional efficiency of these collectors is low compared to powered samplers^26^, the latter were not practicable at these unmanned tundra sites. Holes were drilled through the square bases of the sampler so that long nails could be inserted deep into the soil to anchor them in place. In addition, the basal flange was weighted down by rocks. In June 1972 (late winter), the Tauber samplers were filled in the field with up to 2 cm of glycerol, topped up with several centimetres of distilled water. Glycerol was chosen due to the low rate of evaporation, low toxicity, and low freezing point of glycerol-water solutions of up to −38 °C^34^. A few drops of phenol were added to each sampler to inhibit algal growth and to discourage animal ingestion. In the August 1972 (late summer) expedition, the collection liquid was decanted into numbered polyethylene bags and sealed. For the second year of pollen collection, the collection liquid was replaced with ~1.5 to 2 cm of ethylene glycol (anti-freeze) and ~1 cm of distilled water to bring the effective freezing point down to about −50 °C for the winter of 1972–73, to be collected in August 1973. The phenol added to each sampler to inhibit bacterial or fungal pollen decay proved capable of partially dissolving the polyethylene collecting bags used in the field within a few weeks. Double-bagging and storage in a freezer prevented sample loss within the laboratory.

### Benninghoff or ‘Tuffy’ samplers

At each site, a static, less stable near-surface pollen collector modified from Benninghoff^16^ was placed ~30 cm apart from Tauber sampler TAU1 near the lakeshore, usually dug into a sheltered hollow, which may have resulted in the reduced collection of some pollen compared to more exposed and more stable Tauber samplers. The sampler involved a funnel containing a multi-filament nylon pan-scourer (‘Tuffy’ brand) to mimic the collection surfaces of bryophytes and lichens. This was placed in a plastic collection jar which was partially buried in the soil to provide stability. The collection jar had a nominal height of 12 cm, and the top diameter of the jar was 8 cm. The funnel was placed inside the jar, inhibiting evaporation of the collection liquid and entry of insects. The nylon scourer was positioned on top of the jar and anchored through metal paper clips that were wired securely through holes in the jar wall. The pan scourer was coated with glycerol to catch horizontally moving pollen and spores and the precipitation-scavenged ‘rain out’ component^27^, which has proved elsewhere to be a vital aspect often missed by roofed collectors^35^. Field sampling used distilled water spray-washing of the scouring pad and transferral of the glycerol-pollen suspension and any liquid in the container into a labelled polyethylene bag. Phenol was added to reduce pollen deterioration. Due to the corrosive property of the phenol-glycerol-water mixture, the samples were double-bagged to reduce loss of material. The scouring pad was dipped in glycerol and re-emplaced in the jar for the next sampling season.

### Moss and lichen polsters

Numerous living moss and lichen polsters were preferentially collected at each site in the vicinity of Tauber sampler TAU1 or when the quantity or variety of polster taxa were insufficient, they were collected farther afield. This provided a homogenized longer record of pollen deposition, covering several years to several decades^36,37^. The polster samples were collected to compare the pollen data with that of the Tauber and Benninghoff samplers for a majority of these sampling sites. Duplicate polster samples of different moss and lichen taxa were taken at the same spot throughout the study region to test for differential pollen trapping. Identification of the moss and lichen taxa was aided by taxonomists at the herbarium of the University of Colorado in Boulder. The moss and lichen polsters are stored in H. Nichols’ private collection.

### Vegetation description

The vegetation at each sampling location was documented by numerous 35 mm colour photographs, Super 8 video and sound recordings, and notebook entries. The brief notes on plant communities on each site were strictly time-limited due to airplane charter logistical factors and airplane waiting time costs. Therefore, extensive vegetation surveys were beyond the scope of this study.

### Sample preparation

Prior to chemical treatment, a concentration step to enrich the pollen and spore content of the samples was performed. The collection liquid of the Tauber and Tuffy samplers stored in the plastic bags was washed through a 250-μm mesh and centrifuged, followed by another wash to remove the phenol. The moss and lichen polsters were sectioned in the laboratory, oven-dried at 35 °C, and the dry weight of the sub-samples was recorded. The sub-samples were washed thoroughly and the pollen suspension screened through a 250-μm mesh and centrifuged. This step was repeated once.

Chemical treatment involved sodium hydroxide treatment, acetolysis, dehydration with ethanol, fuchsin staining, and glycerol suspension^38^. When necessary, the polster samples were treated with hydrochloric and hydrofluoric acids. To calculate the percentage loss of the pollen during chemical treatment^5^, a known amount of *Lycopodium clavatum* tracer spores was added to selected polster sub-samples prior to processing^39^. An aliquot of the pollen-glycerol suspension was transferred to a slide and the weight recorded. Some duplicate pollen samples were prepared from the collection liquid of the Tauber and Tuffy samplers when the original storage polyethylene bag became porous due to the corrosive property of phenol and the bag content leaked into the second container bag. The collection liquids of both bags were treated separately, thus the pollen counts of the ‘duplicate’ samples differ.

### Pollen counting

Routine counting was performed at 500-magnification using a Leitz compound microscope, model LS. Pollen and spore types were usually identified to family or genus level, using H. Nichols’ personal reference collection. The standard pollen sum counted for each sample was 300 pollen grains and spores, which was standard practice at the time. The used pollen sum excludes Cyperaceae, Ericaceae, *Filipendula*, *Lycopodium clavatum*, *Lycopodium selago*, *Polypodium*, *Pteridium*, *Selaginella*, and *Sphagnum*. In samples with lower pollen concentrations, a smaller total was counted. Routinely, the number of palynomorphs was recorded at intermediate pollen sums of 100 and 200 totals to determine the internal consistency of the final 300 pollen count^5,40^. Inconsistent counts resulted in re-sampling and re-counting of the respective sample. Palynomorph numbers on a slide were determined by counting the entire slide if pollen concentration was low or by recording the number of traverses necessary to obtain a specific pollen sum^41^. Some samples were only scanned for rare pollen types and their presence noted.

Trace amounts of ‘exotic’ *Pinus* and *Picea* pollen were regularly registered in the samples. Their occurrence reflects the regional airborne transport from the Boreal Forest of the Taiga Shield into the arctic tundra^25^. As their percentage occurrence was very low compared to the local pollen deposition, meaningful changes in long-distance pollen influx could not reliably be inferred from a normal count. To enhance the low exotic pollen numbers as indicators of wind strength and direction and airmass origin, complete scanning of an entire microscope slide (40×22 mm cover slip area) for the bisaccate pollen of *Pinus* and *Picea* was performed at 200x magnification. These statistically more reliable results have significant value in the interpretation of Holocene pollen profiles where the distance to the former arctic tree-line is unknown.

### Data analysis

Three types of pollen data were prepared: a) the conventional percentage data, excluding locally derived pollen and spore types, b) absolute pollen data, and c) absolute pollen data for scan counts for *Pinus* and *Picea* per treated sample or sampler type^41^. Percentage data were calculated for the intermediate and final pollen counts at 100, 200, and 300 totals. Percentages for all pollen and spore types are based on the basic pollen and spore sum. Palynomorph percentages excluded from the basic pollen sum may reach values above 100%.

Absolute palynomorph numbers of the Tauber and Tuffy samplers and the moss and lichen polsters were determined following the method by Jørgensen (1967)^42^. This method proved to be suitable for arctic and subarctic sediments^5,9,12^. Initially, the palynomorph numbers on a slide (N) were determined for all sampler types by recording the number of microscope traverses (t) scanned to obtain a certain number of pollen grains (n) and the total number of traverses possible (T) on that particular cover slip area (40×20 mm) on the particular microscope at a given power (magnification)^31^:

(1)N=n*T/t(allsamplers).

In the next step, the total number of palynomorphs in the original (sub-)sample (N′′) was determined. For polster samples, the total palynomorph number (N′′) per gram dry weight of the sub-sample was based on the number of pollen grains on a slide (N; equation 1) and the treated sub-sample weight (A) divided by the dry weight of the pre-treated sub-sample (G) and the weight of the aliquot on the slide (i.e., slide weight [a]):

(2)N′′=(N*A)/(G*a)(polstersamples).

For the Tauber and Tuffy samples, the total number of palynomorphs per trap (pre-treated sample [N′′]) was first calculated based on the number of pollen grains on a slide (N; equation 1) and the treated sample weight (A) divided by the weight of the suspension on the slide (i.e., slide weight [a]):

(3)N′′=(N*A)/a(TauberandTuffysamples).

The total number of palynomorphs per sample per cm^2^ (N′′′) was calculated by normalizing the total number of palynomorphs per pre-treated sample (N′′) to the depositional area of the Tauber sampler (19.64 cm^2^) and the Tuffy sampler (50.29 cm^2^), respectively:

(4)N′′′=N′′/Area(TauberandTuffysamples).

In addition to the calculation of the absolute data for *Pinus* and *Picea* from only a fraction of the cover slip, the absolute values were also determined by recording their pollen numbers from an entire slide. These so-called SCAN counts for *Pinus* (SPIN) and *Picea* (SPIC) were transformed into absolute numbers per gram dry weight of the pre-treated sub-sample for polsters (equation 2) and per trap (pre-treated sample) and cm^2^ (sampling area) for Tauber and Tuffy samplers using equations 3 and 4, with the number of microscope traverses (t) equalling the total number of traverses possible (T). Determining the absolute numbers for *Pinus* and *Picea* in a sample by counting a fraction of a slide or an entire slide should yield similar results. However, the absolute data based on both counting methods vary to different degrees as the scan counts better register the uneven pollen distribution in a slide and provide higher, statistically more significant pollen counts.

As the efficiency of the sample recovery has a considerable impact on the validity of the data and may involve a later re-preparation of a sample^32^, the recovery factor as a percentage was calculated for polster samples. Calculation of the percentage recovery (RF) was based on the number of exotic tracer spores recovered per gram of the original dried sub-sample (e) and the known amount of exotic tracer spore added to a gram of the original dried sub-sample (E):

(5)RF=(e/E)*100),

whereby (e) is calculated based on equations 1 and 2:

(6)e=(n*T/t*A/(G*a).

## Data Records

The data set described here is stored at the World Data Center PANGAEA (Data Citation 1, Data Citation 2, Data Citation 3, Data Citation 4, Data Citation 5). A parent data set (Data Citation 1) merges the vegetation description (Data Citation 2), the pollen count data (Data Citation 3, Data Citation 4), and the ten original Excel files (Data Citation 5) and thus provides a single citation and DOI for all data sets (Table 2). The description of the vegetation recorded at each sampling site also contains geographic information of the sites (Data Citation 2).

Due to the complexity of the original data set, only the pollen count data tallied at the final pollen sums for the Tauber and Tuffy samplers (Data Citation 3) and the moss and lichen polsters (Data Citation 4) are available as separate data sets in PANGAEA. These data sets provide geographical site and sample information. Pollen data counted at the intermediate pollen sums, the pollen percentage data determined at the intermediate and final pollen sums, the concentration values for all palynomorphs, the concentration data for *Pinus* and *Picea* determined by scan counts, and the associated parameters required for the calculations are given for the Tauber and Tuffy samplers and moss and lichen polsters in eight of the original Excel files (Data Citation 5). The percentage recovery of the prepared sample is given for the moss and lichen polsters in an additional Excel file. Metadata sheets at the beginning of the Excel workbooks provide detailed sample and site information as well as the letter code for the palynomorphs counted. Site information includes the location name, coordinates, the elevation, and the vegetation type. A summary dataset of site and sample information, the pollen count data determined at the final pollen sum, and the counts for *Picea* and *Pinus* pollen for an entire slide was uploaded to figshare (Data Citation 6).

The ‘Sample ID’ is composed of a site identifier (e.g., S1 for Site 1), the sampling period (e.g., T2=Jun-72), the sampler type followed by an identification number (e.g., TAU_1 ‒ Tauber sampler 1; TFY ‒ Tuffy sampler; POL_1 ‒ polster sample 1). A letter at the end of the Sample ID identifies duplicate samples (e.g., TAU_2A; TFY_A; POL_2A). Sample details such as the different sample weight measurements, the slide area inspected, and the number of counted traverses are given. For polster samples, the number of exotic tracers added to a gram of original sample is listed. The counted and transformed pollen and spore data are presented in separate worksheets (count data, percentage data, and concentration data) and divided into three groupings: trees and shrubs, herbs, and pteridophytes. Within each group, the palynomorphs are listed alphabetically. Pollen numbers are given for standard counts. Some samples were only scanned for rare pollen types and their presence is noted as ‘SCAN’ in the original Excel workbooks and as ‘#1’ in the PANGAEA data sets. The count data and the concentration or absolute values determined for *Pinus* and *Picea* by scanning an entire slide are designated ‘SPIN’ and ‘SPIC’ in the data spreadsheet, respectively. The concentration values are given per gram dry weight of the original sub-sample for lichen and moss polsters and per trap and cm^2^ for the Tauber and Tuffy samplers.

## Technical validation

The field study was designed in a way that all installed samplers could be completely relocated despite the often physiographically monotonous nature of much of the tundra surface. The samplers were set out wherever possible on obvious topographic features (e.g., on the point of an esker jutting out into a lake, by a large peat bank, or even near a wolf den) and were marked in winter with orange flagging tape. The regular spacing between the samplers aided relocation of the Tauber samplers. At all the 34 sites, three internationally-accepted and wind-tunnel tested standard static Tauber pollen samplers^15^ were installed. The samplers were set out prior to and after the local flowering season to capture the complete seasonal deposition of pollen. As these samplers collected only two years of pollen deposition, surface samples of living moss and lichen polsters were collected to represent the previous years or decades of pollen deposition in a natural fibrous collection medium. The collection efficiency of the different bryophytes and lichens forms a comparative check on the standard two-season samplers.

The ‘late’ start of the first sampling season in early June 1972 and its comparatively short duration might appear to have shortened that recording season compared to the longer second sampling season from August 1972‒August 1973. However, the main aim of the study was to register the *Picea* and *Pinus* pollen from the northern sub-arctic woodlands and the arctic tree-line ecotone and any pollen from those taxa growing as tree-islands in the tundra. These ecosystems were snow-covered and not flowering in early June 1972 due to abnormally late arrival of spring so that the entire season was indeed effectively captured. Although some exotic pollen from the central and southern Boreal Forest may have been missed in 1972, they were not the focus of this study. The dominant northerly winds in early June 1972 in Keewatin also suggested that such a southerly influx was negligible. This unusual seasonal contrast with the longer 1973 sampling period had a stronger effect than the slightly foreshortened 1972 collection period. However, the most comprehensive long-distance component is recorded in the polster samples, which represent unquantified multiple years of pollen deposition.

Reliability of the Tauber samplers was high in the uninhabited interior transect where they withstood the winter conditions and were undisturbed by animals, but they were at risk of interference in the Inuit villages (Baker Lake, Eskimo Point, Whale Cove, Chesterfield Inlet, and Repulse Bay), so that they were placed on building rooftops for the second sampling period from August 1972 to August 1973. The change in distance from the local vegetation to the samplers is negligible because the focus of the sampling program in the arctic tundra was to record the long-distance exotic pollen. The DEW-line stations were all located at a distance from native settlements and thus those samplers were generally left undisturbed. The Tuffy samplers’ nylon mesh was however often lost to animals though the collecting vessel half-buried in the soil generally survived these attacks. Unfortunately, this inexpensive sampler’s reliability compared poorly with the robust but expensive Tauber samplers under such challenging conditions.

Standard treatment procedures of the samples and standard pollen identification provided consistency. Internal validation of the counted samples was initially performed through repeat counts of the pollen samples by two analysts of the research team. During routine counting, repeat counts were only performed by a second analyst if the original counts appeared anomalous. Internal variations of the samples were tested by recording pollen numbers at different pollen totals. Inconsistent counts resulted in re-sampling and re-counting of the respective sample. The original slides are no longer available.

## Usage notes

The pollen data can be downloaded from PANGAEA. The described database is a geographically unique expeditionary data set from the Canadian Arctic that provides environmental evidence from the 1970’s before the current arctic warming occurred and is thus a baseline to which future pollen collections may be compared. The proximity of some sample sites to meteorological stations provides an opportunity to study modern and past pollen production in relation to changing summer temperatures. Vegetation descriptions made at the sampling sites in 1972–73 may provide additional valuable information for future environmental studies in the Canadian Arctic.

## Additional Information

**How to cite this article**: Nichols, H. & Stolze, S. Modern pollen data from the Canadian Arctic 1972–1973. *Sci. Data* 4:170065 doi: 10.1038/sdata.2017.65 (2017).

**Publisher**’**s note**: Springer Nature remains neutral with regard to jurisdictional claims in published maps and institutional affiliations.

## Supplementary Material



## Figures and Tables

**Table 1:** Locations of modern pollen sampling sites in the Canadian Arctic in 1972–73.

**Site**	**Site name**	**Latitude**	**Longitude**	**Elevation (m)**	**Vegetation**	**Sample Collection**		
						**Tauber**	**Tuffy**	**Polster**
**S1**	Nueltin Lake	60°1'59.97"N	99°51'0.03"W	278	LW	Aug-73	Aug-73	Aug-72
**S2**	Nueltin Lake	60°9'60.00"N	99°44'0.00"W	288	E	Aug-73	Aug-73	Aug-72/73
**S3**	Nueltin Lake	60°21'0.00"N	99°42'0.00"W	280	E	Aug-73	Aug-73	Aug-73
**S4**	Nueltin Lake	60°25'60.00"N	99°38'0.00"W	288	E	Aug-73	Aug-73	Aug-72/73
**S5**	Nueltin Lake	60°34'0.00"N	99°24'0.00"W	277	E	Aug-73	Aug-73	Aug-72
**S6**	Nueltin Lake	60°39'48.88"N	99°22'15.46"W	291	E	Aug-73	Aug-73	Aug-72
**S7**	Nueltin Lake	60°47'45.49"N	99°13'35.28"W	277	E	Aug-73	Aug-73	Aug-72
**S8**	Sealhole Lake	60°49'51.21"N	98°58'46.91"W	279	E	Aug-73	Aug-73	Aug-72
**S9**	Sealhole Lake	60°51'23.75"N	98°42'19.14"W	267	E	Aug-73	Aug-73	Aug-72/73
**S10**	Ennadai Lake	61°7'47.31"N	100°53'30.32"W	314	E	Aug-72/73	Aug-72/73	Jun-72
**S11**	Unnamed lake	61°37'9.08"N	100°7'28.56"W	310	E	Aug-72/73	Aug-72/73	Jun-72/Aug-72
**S12**	Unnamed lake	62°1'60.00"N	99°44'0.00"W	280	T	Aug-72/73	Aug-72/73	Jun-72/Aug-72
**S13**	Yathkyed Lake	62°46'5.53"N	98°21'59.70"W	141	T	Aug-72/73	Aug-72	Jun-72
**S14**	Unnamed lake	63°16'44.62"N	97°45'32.85"W	127	T	Aug-72/73	Aug-72	Jun-72
**S15**	Unnamed lake	63°42'7.33"N	97°36'58.70"W	128	T	Aug-73		Jun-72
**S16**	Unnamed lake	63°58'60.00"N	96°30'0.00"W	63	T	Aug-72/73	Aug-72/73	Jun-72
**S17**	Baker Lake	64°18'54.09"N	96°0'33.56"W	14	T	Aug-72/73	Aug-72/73	Jun-72
**S18**	Tehek Lake	64°41'39.69"N	95°26'49.13"W	126	T	Aug-73	Aug-73	Jun-72
**S19**	Unnamed lake	65°17'39.31"N	95°0'5.56"W	154	T	Aug-73	Aug-73	Jun-72
**S20**	Esker Lake	66°13'0.00"N	92°23'0.00"W	260	T	Aug-73	Aug-73	Aug-72
**S21**	Sandspit Lake	66°54'39.33"N	91°21'6.38"W	290	T	Aug-73		Aug-72
**S22**	Pelly Bay	68°26'13.00"N	89°43'34.00"W	198	T	Aug-73		Aug-72
**S23**	Keith Bay	68°15'19.75"N	88°10'25.34"W	12	T			Aug-72
**S24**	Mackar Inlet	68°18'1.53"N	85°40'28.48"W	324	T	Aug-73		Aug-72
**S25**	Sarcpa Lake	68°33'55.47"N	83°28'53.36"W	285	T	Aug-73		Aug-72
**S26**	Hall Beach	68°45'38.43"N	81°13'35.08"W	0	T	Aug-72/73		Aug-72
**S27**	Longstaff Bluff	68°56'12.23"N	75°17'30.36"W	12	T	Aug-73		Aug-72
**S28**	Dewar Lakes	68°37'40.76"N	71° 7'10.05"W	176	T	Aug-73	Aug-73	Aug-72
**S29**	Cape Christian	70°31'34.71"N	68°17'50.54"W	0	T	Aug-73	Aug-73	Aug-72/73
**A**	Eskimo Point	61°5'56.36"N	94°4'4.55"W	6	T	Aug-72		
**B**	Whale Cove	62°10'18.90"N	92°34'42.22"W	7	T	Aug-73		
**C**	Rankin Inlet	62°48'37.61"N	92°5'9.83"W	20	T	Aug-72/73		
**D**	Chesterfield	63°20'41.59"N	90°43'45.82"W	7	T	Aug-72	Aug-72	
**E**	Repulse Bay	66°31'23.97"N	86°14'24.99"W	19	T	Aug-72/73	Aug-72	
Ecosystems include Lichen Woodland (LW), Arctic Tree-line Ecotone (E), and Tundra (T). The time of pollen collection from the Tauber and Tuffy samplers as well as the moss and lichen polsters is listed (Jun-72=June 1972; Aug-72=August 1972; Aug-73=August 1973).								

**Table 2:** Aggregate information of the data derived from the modern pollen sampling in the Canadian Arctic in 1972-73, listing sample details, methods, data files, and links to the data repository PANGAEA.

**Source**	**Samples**	**Sample number**	**Temporal range 1**	**Temporal range 2**	**Data output**	**Data file**
http://doi.pangaea.de/10.1594/PANGAEA.869619 http://doi.pangaea.de/10.1594/PANGAEA.869614	Tauber and Tuffy samplers	205	Annual pollen deposition	Flowering seasons of 1972 and 1973	Count pollen data	Nichols-Stolze_TAU&TFY_CountData.xlsxNichols-Stolze_2017_TauTuff.tab
http://doi.pangaea.de/10.1594/PANGAEA.869619	Tauber and Tuffy samplers	205	Annual pollen deposition	Flowering seasons of 1972 and 1973	Percentage pollen data	Nichols-Stolze_TAU&TFY_PercentageData.xlsx
http://doi.pangaea.de/10.1594/PANGAEA.869619	Tauber and Tuffy samplers	205	Annual pollen deposition	Flowering seasons of 1972 and 1973	Absolute pollen data	Nichols-Stolze_TAU&TFY_AbsoluteData.xlxs
http://doi.pangaea.de/10.1594/PANGAEA.869619	Tauber and Tuffy samplers	205	Annual pollen deposition	Flowering seasons of 1972 and 1973	Count and absolute *Pinus* and *Picea* pollen data	Nichols-Stolze_TAU&TFY_SPIN&SPICData.xlsx
http://doi.pangaea.de/10.1594/PANGAEA.869619 http://doi.pangaea.de/10.1594/PANGAEA.869616	Moss and lichen polsters	300	Multi-annual pollen deposition		Count pollen data	Nichols-Stolze_POL_CountData.xlsx Nichols-Stolze_2017_MossLichen.tab
http://doi.pangaea.de/10.1594/PANGAEA.869619	Moss and lichen polsters	300	Multi-annual pollen deposition		Percentage pollen data	Nichols-Stolze_POL_PercentageData.xlsx
http://doi.pangaea.de/10.1594/PANGAEA.869619	Moss and lichen polsters	300	Multi-annual pollen deposition		Absolute pollen data	Nichols-Stolze_POL_AbsoluteData.xlsx
http://doi.pangaea.de/10.1594/PANGAEA.869619	Moss and lichen polsters	300	Multi-annual pollen deposition		Count and absolute *Pinus* and *Picea* pollen data	Nichols-Stolze_POL_SPIN&SPICData.xlsx
http://doi.pangaea.de/10.1594/PANGAEA.869619	Moss and lichen polsters	300	Multi-annual pollen deposition		Percentage sample recovery	Nichols-Stolze_POL_SampleRecoveryData.xlsx
http://doi.pangaea.de/10.1594/PANGAEA.869619 http://doi.pangaea.de/10.1594/PANGAEA.869617	Vegetation description of the sampling sites	34	Plant taxa	June 1972–August 1973	Vegetation and environmental settings	Nichols-Stolze_Vegetation1972-73.xlsx Nichols-Stolze_2017_sites.tab
